# Chylothorax and chylopericardial tamponade following lobectomy and lymphadenectomy: a rare presentation

**DOI:** 10.1186/s13019-023-02126-3

**Published:** 2023-01-16

**Authors:** Guofei Zhang, Tianshu Liu, Chengxiao Liang

**Affiliations:** 1 Department of Thoracic Surgery, The Second Affiliated Hospital of the Zhejiang University School of Medicine, 88 Jiefang Road, Hangzhou, 310009 China; 2 Department of Surgery, Zhejiang Hospital, # 12 Lingyin Road, Hangzhou, 310013 China

**Keywords:** Chylothorax, Chylopericardium, Cardiac tamponade, Lung cancer, Lobectomy, Lymphadenectomy

## Abstract

**Background:**

Although postoperative chylothorax following lung cancer surgery is rare, it is a recognized complication in 0.25–3% of patients. However, cases of cardiac tamponade caused by chylopericardium after lung cancer surgery are extremely rare.

**Case presentation:**

We describe hitherto unreported sequelae of chyle leak following lobectomy and systematic mediastinal lymph node dissection (SLND) causing pericardial tamponade and cardiovascular compromise. The patient was successfully treated with minimally invasive surgical repair and ligation. We also discuss the development of chylopericardium as a potential complication of lobectomy and SLND.

**Conclusions:**

The anatomical characteristics of the thoracic duct warrant special attention in postoperative chyle leak management in patients who undergo definitive mediastinal lymph node dissection. Surgeons should be aware that chylopericardium is a rare but potential complication of lobectomy and SLND as it may help with early diagnosis, management, and prevention of cardiac tamponade.

**Supplementary Information:**

The online version contains supplementary material available at 10.1186/s13019-023-02126-3.

## Background

While postoperative chylothorax after lung cancer surgery is rare, postoperative chylopericardium is an extremely rare but fatal complication that usually occurs after various cardiothoracic procedures [1–3]. Moreover, there are limited reports in the literature on chylopericardium complicating lobectomy and lymphadenectomy. Here, we report a case of acute cardiac tamponade caused by chylopericardium after lobectomy and systematic mediastinal lymph node dissection (SLND) and discuss the causes and treatment of this complication.

## Case presentation

A 54-year-old man presented with a persistent cough for two months without hemoptysis. He was overall healthy but had a smoking history of 30 pack-years. On physical examination, no abnormalities were observed. Chest computed tomography (CT) revealed a 2.5 cm × 2.0 cm mass in his right lower lobe, suggesting malignancy. Bronchoscopy revealed a tumor infiltrating the distal end of the right bronchus intermedius. A pathological diagnosis of squamous cell carcinoma was made based on the biopsy specimen. Abdominal CT, cranial magnetic resonance imaging, and whole-body bone emission CT were performed to rule out distant metastases. Subsequently, the tumor was staged as cT1cN0M0 (IA2) according to the 8^th^ edition of the TNM lung cancer staging system.

**Fig. 1:**
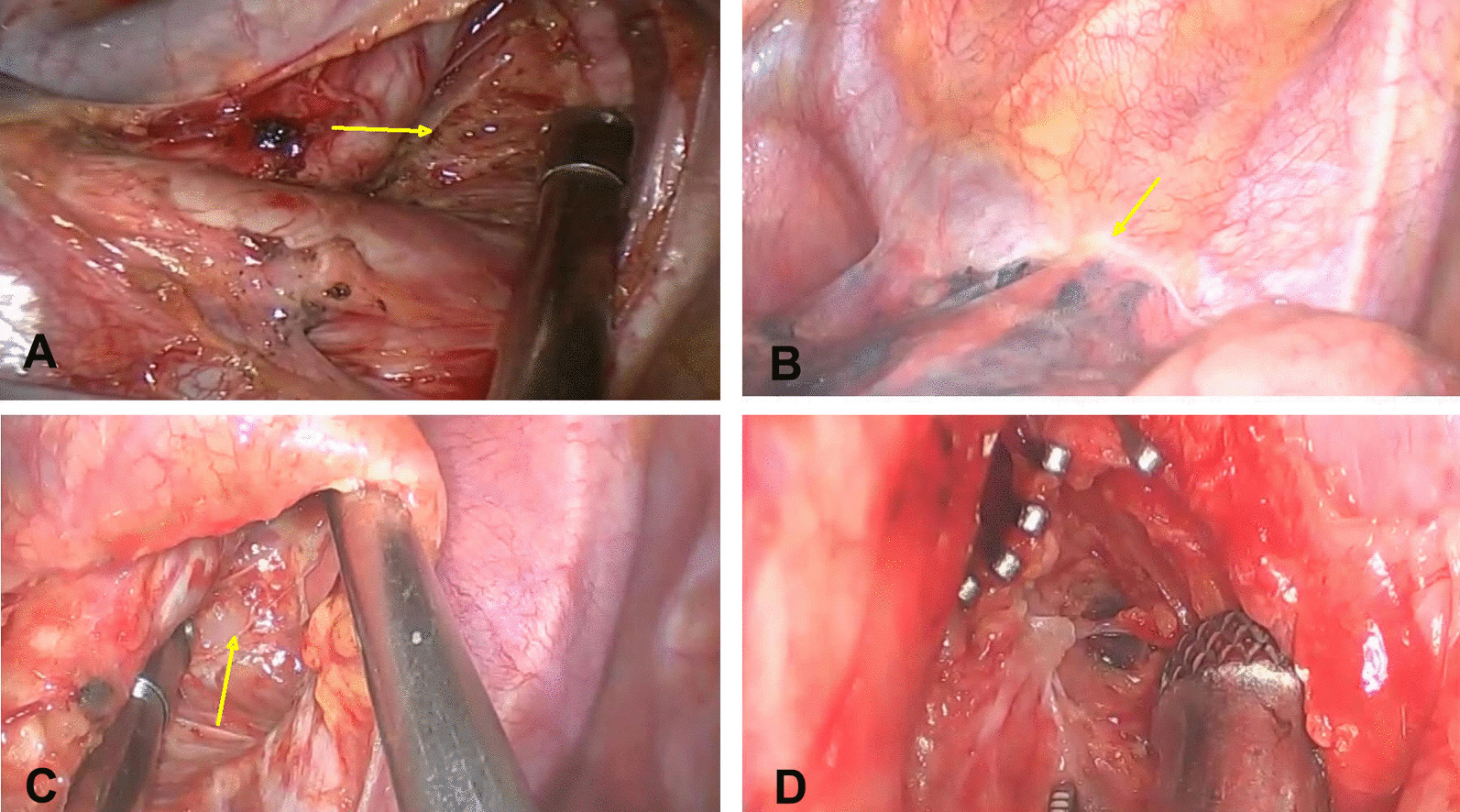
**A** The second and fourth group lymph node dissection (arrow) at the first operation. **B** Operative finding of the second operation. Adhesions have formed below the arch of azygos vein and the upper right lung (arrow). **C** A diffuse chylous leak is identified as originating from the tributary channels at the site of dissection of the station 4R lymph nodes (arrow). **D** Tributary channels are clamped using titanium clips

The patient underwent minimally invasive thoracic surgery. Right lower and middle bilobectomies with mediastinal lymph node dissection were performed using uniportal video-assisted thoracoscopic surgery (VATS) (Fig. 1A). Histopathological examination of the operative specimen revealed squamous cell carcinoma (pT1cN0M0, IA2) of the lung. However, after starting enteral feedingon the first day after surgery, the volume of the pleural fluid drainage increased and changed from serous to an opaque “creamy” fluid, suggesting chylothorax. Although conservative treatment was applied for 5 days, which included total parenteral nutrition and complete cessation of oral intake, the drainage output remained above 600 mL/day.

On the sixth postoperative day, the patient suddenly developed sinus tachycardia and hypotension (systolic blood pressure < 60 mmHg, unresponsive to fluid boluses). A chest radiograph did not showany obvious abnormality; however, a bedside echocardiogram revealed significant diastolic collapse caused by a large pericardial effusion. An emergency pericardiocentesis drained 400 mL of chylous fluid, resulting in dramatic clinical improvement. As the characteristics of the pericardial drainage fluid were like those of the previous pleural drainage fluid, a diagnosis of cardiac tamponade caused by chylopericardium was considered. The pericardial fluid continued to drain at a rate of 600 mL/24 h or more for the next 7 days, while the pleural drainage was less than 100 mL/24 h.

Subsequently, a uniportal VATS examination was performed under general anesthesia through the original incision (Additional file 1: Video 1). Four hours before surgery, the patient was instructed to drink 100 mL of thick cream. Intraoperatively, adhesions had formed below the arch of the azygos vein and the upper lobe of the right lung (Fig. 1B). After the release of the adhesions, a slow chyle leak was detected from the tributary channels at the site of dissection of station 4R lymph nodes (Fig. 1C). The pleuropulmonary adhesions had formed a barrier sequestering the chyle from the free pleural cavity and diverting the chyle drainage into the pericardium through a pericardial defect in the superior part of the pericardium near the right pulmonary artery. No major injury to the thoracic duct was observed. Surgical interventions included clamping the tributary channels using titanium clips (Fig. 1D), ligation of the thoracic duct near the diaphragm, and pericardial fenestration to allow adequate drainage.

Postoperatively, there was no recurrence of pericardial or pleural effusion. The right pleural and pericardial fluid drainage gradually decreased, and the drainage tubes were removed on the fourth day. The patient was discharged on the fifth postoperative day in a stable condition. Chest radiograph showed no abnormalities during his 3-month follow-up period, and his quality of life improved.

## Discussion and conclusions

Chylothorax occurs in 0.25–3% of cases following lobectomy and SLND [2]. However, cases of cardiac tamponade caused by chylopericardium after lobectomy and SLND are rare. This case illustrates that chylothorax and chylopericardium are potential complications of lobectomy and SLND.

Although intrathoracic lymphatic pathways are generally uniform, a rare anatomical variant may have caused the problem in our patient, which explains the lack of precedent for this report [4, 5]. Occasionally, an efferent lymphatic trunk may drain into the thoracic duct within the mediastinum via the right paratracheal lymph nodes [6]. In our patient, the lymphatic trunk was injured during dissection of the station 4R lymph nodes, while the adjacent pericardium was also ruptured by an ultrasound scalpel. The resultant transparent chyle was mistaken for normal fluid, which prevented timely discovery and ligation. On the sixth day after surgery, pleuropulmonary adhesions resulted in sequestration of chyle from the free pleural cavity and diversion of chyle drainage into the pericardium through the pericardial defect. Consequently, the patient developed acute cardiac tamponade. Failure of conservative treatment necessitates surgical interventions such as thoracic duct ligation or clamping [7]. Therefore, as the pericardial fluid drainage did not reduce, we decided to proceed with surgical interventions to prevent the recurrence of cardiac tamponade.

In conclusion, the anatomical characteristics of the thoracic duct warrant special attention in postoperative chyle leak management in patients who undergo definitive mediastinal lymph node dissection. Like other causes of pericardial effusion, chylopericardium may lead to pericardial tamponade. Surgeons should be aware of this rare but potential complication of lobectomy and SLND as it may help in early diagnosis and management.

## Supplementary Information

**Additional file 1. Video 1:** Uniportal VATS mediastinal lymph node dissection (second and fourth groups) at the first operation and surgical intervention for chylopericardium at the second operation. VATS, Video-assisted thoracoscopic surgery.

## Data Availability

The datasets used and/or analyzed during the current study are availablefrom the corresponding author on reasonable request.

## Abbreviations

SLND: Systematic mediastinal lymph node dissection
CT: Computed tomography
VATS: Video-assisted thoracoscopic surgery
